# Quality-of-Life (QOL) during Screening for Phase 1 Trial Studies in Patients with Advanced Solid Tumors and Its Impact on Risk for Serious Adverse Events

**DOI:** 10.3390/cancers9070073

**Published:** 2017-06-26

**Authors:** Sidra Anwar, Wei Tan, Chi-Chen Hong, Sonal Admane, Askia Dozier, Francine Siedlecki, Amy Whitworth, Ann Marie DiRaddo, Dawn DePaolo, Sandra M. Jacob, Wen Wee Ma, Austin Miller, Alex A. Adjei, Grace K. Dy

**Affiliations:** 1 State University of New York at Buffalo, 12 Capen Hall, Buffalo, NY 14260, USA; Sidra.anwar@hotmail.com; 2 Roswell Park Cancer Institute, Elm and Carlton Street, Buffalo, NY 14263, USA; wei.tan@roswellpark.org (W.T.); Chi-Chen.Hong@RoswellPark.org (C.-C.H.); Askia.Dozier@RoswellPark.org (A.D.); Francine.Siedlecki@RoswellPark.org (F.S.); Amy.Whitworth@RoswellPark.org (A.W.); AnnMarie.DiRaddo@RoswellPark.org (A.M.D.); DawnMarie.DePaolo@RoswellPark.org (D.D.); Sandy.Jacob@RoswellPark.org (S.M.J.); Austin.Miller@RoswellPark.org (A.M.); 3 Baylor Scott and White Memorial Hospital, 2401 S 31st St., Temple, TX 76508, USA; sonaladmane@gmail.com; 4 Mayo Clinic, 200 1st St. SW, Rochester, MN 55905, USA; ma.wen@mayo.edu (W.W.M.); Adjei.Alex@mayo.edu (A.A.A.)

**Keywords:** quality-of-life, QOL, EORTC QLQ-C30, FACT-G, MOSSSS, phase 1 cancer trial, oncology trials, immunotherapy, selection criteria, serious adverse events

## Abstract

*Background*: Serious adverse events (SAEs) and subject replacements occur frequently in phase 1 oncology clinical trials. Whether baseline quality-of-life (QOL) or social support can predict risk for SAEs or subject replacement among these patients is not known. *Methods*: Between 2011–2013, 92 patients undergoing screening for enrollment into one of 22 phase 1 solid tumor clinical trials at Roswell Park Cancer Institute were included in this study. QOL Questionnaires (EORTC QLQ-C30 and FACT-G), Medical Outcomes Study Social Support Survey (MOSSSS), Charlson comorbidity scores (CCS) and Royal Marsden scores (RMS) were obtained at baseline. Frequency of dose limiting toxicities (DLTs), subject replacement and SAEs that occurred within the first 4 cycles of treatment were recorded. Fisher’s exact test and Mann-Whitney-Wilcoxon test were used to study the association between categorical and continuous variables, respectively. A linear transformation was used to standardize QOL scores. *p*-value ≤ 0.05 was considered statistically significant. *Results*: Baseline QOL, MOSSSS, CCS and RMS were not associated with subject replacement nor DLTs. Baseline EORTC QLQ-C30 scores were significantly lower among patients who encountered SAEs within the first 4 cycles (*p* = 0.04). *Conclusions*: Lower (worse) EORTC QLQ-C30 score at baseline is associated with SAE occurrence during phase 1 oncology trials.

## 1. Introduction

Phase 1 oncology trials are mainly designed to evaluate the toxicity and pharmacokinetic profiles of investigational agents in order to determine the appropriate dose for subsequent phase 2 testing. Phase 1 trial participants typically have advanced cancer for which standard therapies are either not available or have been exhausted. Though toxic deaths are rare at ~0.5% [1,2], nonfatal serious toxicities may often be encountered. Overall nonfatal serious grade 3 or 4 treatment-related toxicities occurred in approximately 10% of 6474 patients who participated in phase 1 clinical trials reported between 1991–2002 [2]. More recently, phase 1 trials involving molecular targeted agents similarly have an estimated rate of grade 3 or 4 toxicity between 10–15% [3,4].

Much research has been dedicated towards being able to select the “fittest” of the oncologic population for early phase clinical trials. This is not only because of the magnitude of possible toxicities these patients face but also because replacement of patients during the dose-escalation phase, either due to early clinical deterioration or non-treatment related serious adverse events (SAEs), is a common logistical issue that prolongs the study duration due to the nature of dose-escalation study designs. As a result of prior research, organ function, tobacco use and performance status levels have been identified as prognostic factors for toxicity independent of dose administered, while lactate dehydrogenase (LDH) levels and performance status are prognostic factors for survival [5,6]. However, while patient performance status and organ function data are routinely used to determine eligibility for phase 1 study involvement, a recent retrospective review showed a 50% SAE rate in cycle 1 among patients participating in phase 2 trials of molecularly-targeted agents [7]. This demonstrates a gap in our current process for optimizing patient selection to minimize non-treatment related AEs.

The Royal Marsden prognostic score (RMS) was developed in a British center after a retrospective review of 212 phase 1 patients identified LDH, number of metastatic sites and hypoalbuminemia as independent negative prognostic factors for overall survival [8]. It was subsequently reported to be helpful in prospectively evaluating a selected cohort of phase 1 patients [9]. However, RMS is still limited by its crude prognostication ability and its impact on reducing recruitment to phase 1 studies, and thus it is not incorporated routinely in screening procedures for inclusion of patients in phase 1 trials [10].

The Charlson Comorbidity Index (CCI) is another well-validated measure, developed from a longitudinal study of over 500 patients. It assigns a weighted score for certain medical conditions that co-exist for each patient that affect overall mortality [11]. A regression model was created that can predict the occurrence of SAE for patients during the first cycle of phase 2 trials based on a albumin, LDH, number of target lesions, age, performance status and CCI score [7]. However, while CCI has been extensively applied in health services research in cancer patients, its utility in predicting short-term outcomes seems to be more limited [12,13,14].

Quality-of-life (QOL) outcome measurements have an established role in oncology clinical trials, including the drug approval process, especially when survival outcomes compared to standard of care are not significantly different [15,16]. It has also been shown that among general cancer patients receiving cytotoxic chemotherapy, patients with high QOL lived significantly longer than patients with low QOL, particularly in patients with metastatic disease [17]. Despite its potential prognostic role, there is lack of data on the utility of QOL evaluation for patient selection in phase 1 oncology clinical trials.

Social support, though often overlooked by physicians, seems to also play an instrumental role in outcomes for cancer patients. Cancer patients with poor social support suffer from increased rates of depression [18] and decreased compliance with treatment [19], making them more susceptible to disease progression [20] and increased mortality [21].

We thus aimed to prospectively evaluate whether assessment of QOL and social support at time of study screening can be a tool in evaluating patient fitness and risk for SAEs and subject replacement in phase 1 studies.

## 2. Methods

Between September 2011 to August 2013, patients ≥18 years of age with histologically or cytologically confirmed solid tumors were approached for participation in this prospective, observational study at the time of screening for any of 22 phase 1 clinical trials, excluding phase 1 trials that involve regional therapies such as radiation, surgery or photodynamic therapy, at Roswell Park Cancer Institute (Supplementary Table S1). Patients unable to read or understand English were excluded. Informed consent to this study meeting Federal and Institutional requirements was obtained from each patient prior to registration. Institutional review board approval was obtained for this study.

QOL and social support questionnaires were administered at baseline anytime between screening for the phase 1 trial and before the first treatment day. Questionnaires were thereafter administered on day 1 of each subsequent treatment cycle until day 1 of cycle 4 if patient was still enrolled in the therapeutic study. The three questionnaires used were Functional Assessment of Cancer Therapy-General (FACT-G) [22], European Organization for Research and Treatment of Cancer Quality of Life Questionnaire-Core 30 (EORTC QLQ-C30) version 3 [23] and Medical Outcomes Study Social Support Survey (MOSSSS) [24]. RMS and CCI were determined at baseline.

Dose-limiting toxicities (DLTs) as defined according to the respective treatment study patient enrolled to, need for patient replacement in the actual interventional phase 1 trial, and all SAEs that occurred within the first four cycles were collected. SAE is defined as any CTC version 4 grade 3 or higher toxicity, regardless of treatment attribution. All consented patients with available LDH levels at baseline for determination of RMS were included in the relevant statistical analysis. Toxicity data, specifically protocol-defined DLTs and SAEs were prospectively collected from the weekly phase 1 safety meeting minutes and verified with chart review.

### Statistical Considerations

A linear transformation was used to standardize the raw QOL score, so that scores range from 0 to 100, with 100 representing the highest level of functioning possible [8,25]. Based on informal review of published data, up to 25% of patients enrolled in phase 1 studies had to be “replaced” during dose-escalation phase. Thus for sample size of 100 patients accrued to the study, there will be about 80% power at a 0.05 significance level to detect minimum odds ratio of 1.9 for patient observations one standard deviation from the mean QOL score.

Descriptive statistics such as frequencies and relative frequencies were computed for categorical variables. Numeric variables were summarized using simple descriptive statistics such as the mean, standard deviation, median, range, etc. Mann-Whitney-Wilcoxon test was used to test significant differences between different groups for numeric variables. Fisher’s exact test was used to test significant differences between different groups for categorical variables. The estimated overall survival distributions were obtained using the Kaplan-Meier method. Using this distributional estimate, summary descriptive statistics such as the median survival and a 95% confidence interval of the median survival were obtained. Statistical assessment of observed differences in the survival distributions of different groups of interest was done using the log-rank test. The Cox proportional hazards model was used to assess the effect of numerical variables on survival analyses. All tests were two-sided and tested at a 0.05 nominal significance level. SAS version 9.4 statistical software (SAS Inc., Cary, NC, USA) was used for all statistical analyses.

## 3. Results

### 3.1. Demographics

A total of 104 patients consented to this study. 92 patients had LDH level drawn at baseline for RMS calculation. At least one baseline questionnaire was completed by all 92 patients who consented to this study and met eligibility for data analysis. Mean age of the patients was 61 years, 41% were males. The majority of the malignancies were of Gastrointestinal origin (42%) followed by the respiratory system (24%) (See patient demographics in Table 1). The majority of patients (72%) were enrolled in trials that included a molecular targeted therapy agent.

### 3.2. QOL and Social Support Analysis

Median days between baseline QOL surveys and first day of study treatment was 8 days (four patients started treatment between 35–53 days after baseline QOL surveys were obtained). As expected, 60% of patients were unable to complete QOL surveys beyond cycle 2 as they were discontinued from the corresponding interventional treatment study for standard reasons. Nonetheless, there does not appear to be any longitudinal change in EORTC QLQ-C30 or FACT-G scores (Table 2). There was a statistically significant increase in MOSSSS by the third cycle of treatment compared to baseline (mean score 91 vs. 87, *p* = 0.041) (Table 2).

### 3.3. Risk of Replacement

Out of the 92 patients enrolled, only 12 patients were replaced, thus underpowering the study to detect whether any of our variables were associated with risk of subject replacement. Across all studies analyzed, the QOL and social support questionnaires were unable to predict subject replacement during the DLT-defining period (Table 3). When analysis was confined to the seven trials wherein subject replacement occurred (12 out of 47 patients enrolled in the specified trials, see Supplementary Table S2 for study details), there was a statistically significant difference in baseline MOSSSS, with a higher median score of 98 (range 79–100) in patients, representing better perceived social support, who were replaced compared with median of 90 (range 20–100) in patients who were not replaced (*p* = 0.041) (Table 4).

### 3.4. Risk of SAE

Twenty two (22) out of 92 patients (24%) encountered SAEs over 24 separate occasions, majority of which occurred during the first cycle. Nineteen (19) of the 22 patients encountered SAEs during cycle 1 therapy, two of whom had another separate SAE in cycle 2 or 3 of treatment. Out of these 24 episodes, 10 were deemed treatment-related, six due to disease progression, and eight due to concomitant medical conditions, including the diagnosis of massive GI bleed in a screen-eligible patient which occurred prior to starting cycle 1 day 1 of therapy. Baseline EORTC QLQ-C30 scores were found to be significantly different between patients who incurred SAE within the first four cycles of therapy and patients who did not encounter SAE within the first four cycles of therapy (*p* = 0.044), with patients who had lower baseline score encountering more SAEs (Table 3). In contrast, baseline FACT-G and MOSSS were unable to predict risk of SAE while enrolled in the interventional clinical trial (Table 3). When EORTC QLQ-C30 scores were further analyzed, none of the individual components however, i.e., Global health status, functional scales and symptom scales were significantly different amongst SAE vs. non-SAE groups (Supplementary Table S4).

### 3.5. Other Outcomes

**Figure 1:**
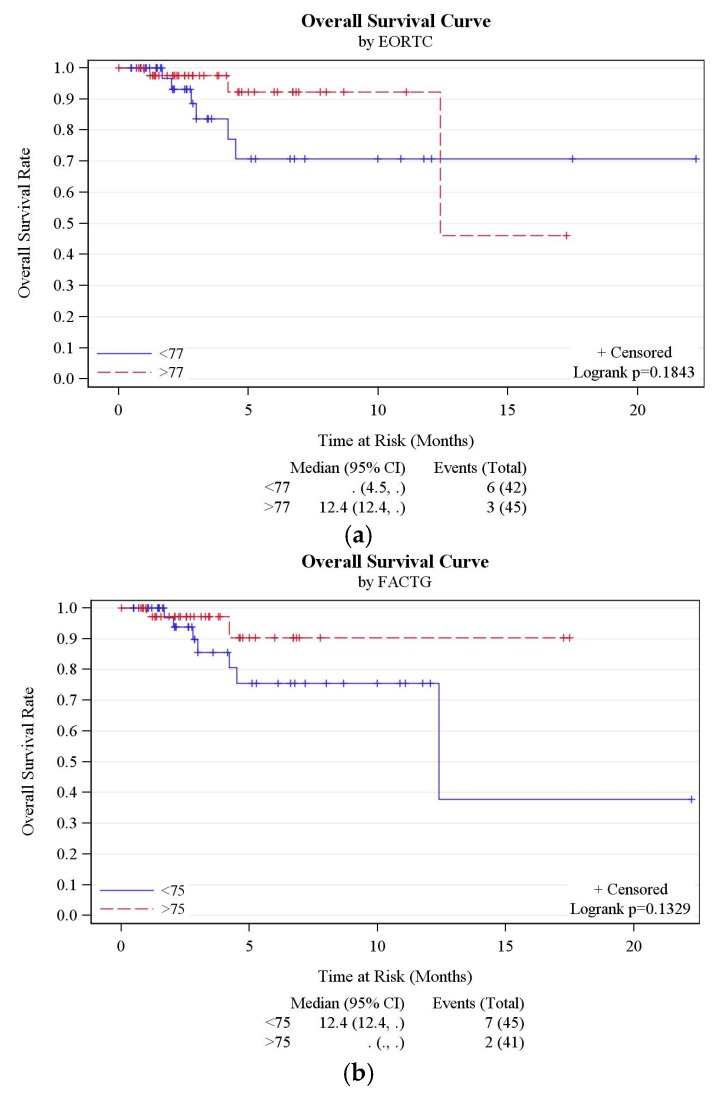

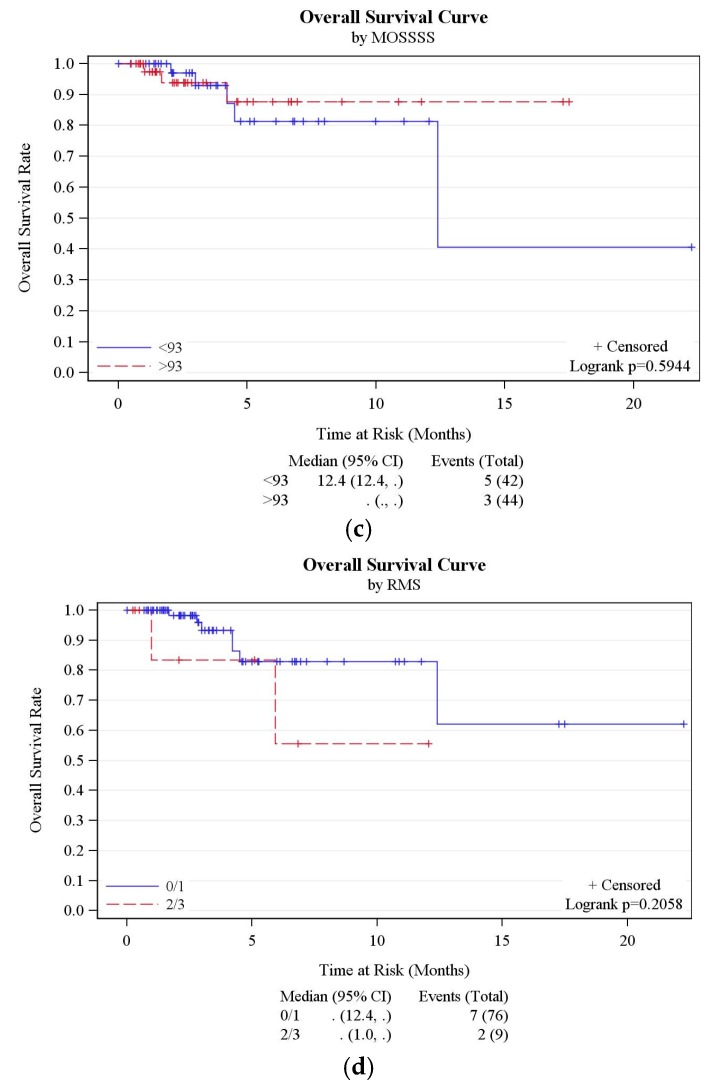
(**a**) Overall survival curve by EORTC QLQ-C30. (**b**) Overall survival curve by FACTG. (**c**) Overall survival curve by MOSSSS. (**d**) Overall survival curve by RMS.

The risk of encountering DLTs and the rate of successful completion of cycle 1 of chemotherapy was not successfully predicted by baseline EORTC QLQ-C30, FACT-G or MOSSSS (Table 3). There was also no correlation between baseline scores and overall survival (Figure 1a–c and Table 5).

### 3.6. RMS Analysis

There was no significant difference in baseline QOL scores according to RMS across the different QOL questionnaires (Table 3). Furthermore, there was no significant difference in number of subject replacements, rate of completion of cycle 1 of chemotherapy, SAE or DLT occurrence between patients with baseline RMS 0/1 versus 2/3 (Supplementary Table S3). There was no correlation with overall survival seen in this cohort of phase 1 trial patients (Figure 1d and Table 5).

## 4. Discussion

While previous research has highlighted the longitudinal prognostic impact of QOL in phases 1–3 trials [26,27,28], this study is the first to demonstrate that baseline QOL scores using a validated tool such as the EORTC QLQ-C30 questionnaire may independently provide an objective measurement to evaluate the risk of incurring SAE for each individual patient during phase 1 trial participation. Differences in the individual components of the EORTC QLQ-C30 questionnaire obtained by the patients were not substantial on their own, but when a cumulative score was obtained for QOL, it was able to achieve statistical significance leading to the conclusion that patients with better median QOL scores incur SAEs less frequently as compared to those with lower scores.

As the EORTC QLQ-C30 questionnaire measures somatic and psychological symptoms, functional status and overall health of the individual it would be reasonable to assume a patient with lower scores is more likely to have worse mental and physical disease burden and demonstrate abnormalities at the biochemical level such as increased Interleukin-2 and Interleukin-6 levels, anemia and hypoalbuminemia, all which are known to contribute towards poor QOL in cancer patients [29,30,31,32,33,34]. These factors combined, along with many others likely predisposes such individuals to SAEs with experimental chemotherapy.

This study found that unlike the EORTC QLQ-C30, the FACT-G QOL questionnaire was not able to stratify patients according to risk for SAE during the trial. The reason may be that although both questionnaires are validated measures of QOL in cancer patients, there are significant differences between their structure, social domain questions and their overall tone [35]. As an example, FACT-G asks patients to reflect on their thoughts and feelings whereas the QLQ-C30 questions focus on more objective aspects of functioning [35]. Therefore, despite considerable overlap, neither of these two QOL questionnaires can be replaced by the other, nor can a direct comparison between their results be made [36].

Another interesting observation is that MOSSSS score was higher by cycle 3 compared to baseline. One may hypothesize that this indirectly reflects that patients with better social support are more likely to stay on study treatment, particularly with phase 1 studies wherein there are generally more research-related tests and procedures involved during the first one or two cycles of treatment. However, this is not supported by our other observation that MOSSSS scores were higher in the group of patients who were replaced compared to patients who were not replaced in the seven phase 1 trials that incurred subject replacement. One possibility is that a patient with comparatively lower score may look upon the care team itself as an important source of social support and thus remain in the study even if non-DLT toxicities were encountered or additional research-related tests are required to be repeated, whereas patients with a higher score maybe negatively influenced in such circumstances by their caregivers to withdraw from a study early.

We believe this study provides rationale for clinicians to consider stratifying potential enrollees into phase 1 oncologic trials according to their baseline QOL. Obtaining QOL scores is possible in a timely manner, averaging 11 min with most patients not requiring assistance [37], and is typically less burdensome to patients than blood draws or CT scans and also more cost-effective. The need for additional measures for patient inclusion in phase 1 trials is highlighted by the fact that despite strict criteria involving performance status, organ function, and LDH levels built into eligibility requirements for phase 1 clinical trials in oncology, there continues to be a considerable degree of early trial discontinuation and patient replacement during phase 1 trials (16% in the series reported by Olmos et al. [10]). More recently, a simplified risk score was proposed to identify patients at risk for early discontinuation prior to study enrollment to address this logistical issue [38]. Unfortunately, the proposed model, according to the authors, will still indiscriminately exclude seven patients for every three patients accurately excluded while early discontinuation rates would remain >10%. This fact thus deters widespread adoption of this metric in patient selection. In comparison, our pilot study aimed to evaluate the use of QOL as a tool to identify patients at risk for incurring toxicities, SAEs and early discontinuation from phase 1 trials.

Once baseline QOL scores are obtained, those with particularly dismal QOL scores should not be considered candidates for the trials, in the same way traditional criteria such as organ function might exclude patients from experimental chemotherapy as the risks greatly outweigh the benefit in these cases. Physicians should proceed cautiously with other patients with moderately low scores. Patients should be counselled as to the increased risks of SAE occurrence and prior to enrollment in any trial they should have aggressive symptom management either by the oncologist or a specialist in palliative medicine with the aim of improving QOL before experimental chemotherapy is begun. Specialist follow up should continue throughout the trial. Discussion of baseline QOL scores and its implications should be incorporated into the informed consent and decision-making process with cancer patients.

Our intent is not to promote another barrier or exclusion from phase 1 trials based on QOL scores. Indeed there are good reasons for all cancer patients to seek participation in well-designed clinical treatment trials [39], including phase 1 trials particularly if they have a realistic probability to derive some benefit despite anticipated adverse events and drug toxicities. While benefits of trial participation traditionally range from being exposed to state-of-the-art treatments [40], to being under the care of physicians participating in clinical trials who, it has been suggested, take better care of all of their patients [41], recent studies confirm that prognosis for phase 1 patients appears to have improved. Meta-analysis of phase 1 studies sponsored by the Cancer Therapy Evaluation Program from 1991 to 2002 revealed overall response and disease stability rates of 10.6% and 34.1%, with overall toxic death rate remaining low (0.49%) in 11,935 participating patients [42]. While survival data is difficult to interpret due to the heterogeneous patient samples involved, it is reassuring that analysis of treatment outcomes in contemporary phase 1 oncology trials shows these trials to be safe and associated with clinical benefit in greater than 40% of patients [42,43], with median survival of 8.7 months [43]. By helping physicians better identify patients at an increased risk for SAEs during the trial period, the chances for successful patient accrual, less subject replacements and maximum clinical benefit to the participants is greatly increased, all of which are key requirements for any trial.

This study has some limitations. Overall this study features a small cohort of patients and the results need to verified on a larger scale. It did not meet the hypothesis that QOL scores are able to predict occurrence of DLTs or subject replacement. One reason may be due to the fact that it is underpowered to detect differences based on the small sample size since out of 92 patients included in the analysis, only 12 were replaced in the overall cohort of patients. More data also needs to be gathered to validate these findings and to model a predictive score for decision algorithms.

While the implications of these findings provide rationale to incorporate QOL assessment in the design and development of phase 1 clinical trials, additional investigation is warranted to validate the role of QOL in patient selection criteria for Phase 1 cancer trials. Future studies may involve dividing QOL scores into ranges that can define high/average or low scores and thus allow patients to be grouped accordingly. This practice would be able to provide clearer guidelines to physicians when making clinical decisions. Prospective research should also focus on the impact of interventions such as early involvement of palliative care in the management of these patients to help diminish the risk of SAEs and potentially early discontinuation from the treatment trial. As the physician-patient relationship has also been shown to affect a patient’s QOL [44,45,46], screening for patient satisfaction with their healthcare professional may be able to identify a potentially modifiable contributor towards poorer QOL.

## 5. Conclusions

Low QOL scores using the EORTC QLQ-C30 questionnaire at baseline is associated with SAE occurrence in patients who go on to receive experimental treatment in phase 1 oncology trials. Clinicians should consider incorporating QOL into screening measures used to determine patient eligibility for these trials. For individuals with worse QOL, the underlying major contributors should be identified and efforts should be made to improve their QOL prior to enrollment. Interventions that may be helpful include improving the quality of physician-patient interactions and relationship, aggressive symptom management and early involvement of palliative care to help mitigate risk for SAEs.

## Figures and Tables

**Table 1:** Demographics of the 92 patients.

Patient Demographics			
AGE (years)	Range: 30–84	Mean: 61.2	Median: 63
GENDER	MALE	38	41%
	FEMALE	54	59%
CANCER	Gastrointestinal tract	39	42%
	Respiratory tract	22	25%
	Gynecological	10	11%
	Sarcoma	8	9%
	Breast	4	4%
	Head + Neck	3	3%
	Endocrine	3	3%
	Lymphoma	2	2%
	Skin	1	1%

**Table 2:** EORTC QLQ C-30, FACT-G and MOSSSS scores throughout the duration of the study.

Questionnaire	Event Identifier	N	N *	Mean	Standard Deviation	Median	Min	Max	*p*-Value Compared with Baseline Score)
EORTC QLQ C-30	BASELINE	92	87	75.3	14.4	77.2	42.5	96.9	-
	VISIT1	87	61	74.6	13.7	77.8	37.5	95.0	0.563
	VISIT2	61	34	78.9	13.6	80.1	43.1	97.5	0.593
	VISIT3	34	23	78.1	16.5	83.6	38.3	97.5	0.191
	VISIT4	26	13	79.7	11.6	80.6	53.3	96.1	0.654
FACT-G	BASELINE	92	86	73.7	14.2	75.0	28.7	94.4	-
	VISIT1	87	61	74.8	13.4	75.9	31.5	94.2	0.166
	VISIT2	61	34	75.3	14.9	74.9	29.6	98.1	0.293
	VISIT3	34	23	79.6	13.9	77.9	50.0	99.0	0.094
	VISIT4	26	13	78.8	10.9	82.7	60.2	98.1	0.420
MOSSSS	BASELINE	92	86	87.0	17.0	93.4	19.7	100	-
	VISIT1	87	60	85.8	19.2	97.4	19.7	100	0.878
	VISIT2	61	34	89.9	15.5	96.7	22.4	100	0.073
	VISIT3	34	23	90.8	11.7	97.4	59.2	100	0.041
	VISIT4	26	13	90.8	13.4	96.1	56.6	100	0.440

N: Number of total patients for each cycle. N *: number of patients who adequately filled out the questionnaires. FACT-G: Functional Assessment of Cancer Therapy-General Score. EORTC: European Organization for Research and Treatment of Cancer QLQ-C30 Score. MOSSSS: Medical Outcomes Study Social Support Survey Score.

**Table 3:** Baseline questionnaires and their respective outcomes.

Questionnaire	Variable	Level	N Obs *	N **	Mean ^	Standard Deviation	Median ^	Min	Max	*p*-Value
EORTC QLQ C-30	DLT	Yes	6	5	69.7	11.1	67.5	60.0	87.8	0.245
		No	86	82	75.6	14.6	78.8	42.5	96.9	
	CYCLE1 completed	Yes	62	59	76.8	13.4	80.7	42.5	94.7	0.218
		No	30	28	72.1	16.1	73.2	43.1	96.9	
	Replacement	No	80	77	75.7	13.5	77.2	42.5	96.4	0.867
		Yes	12	10	72.3	20.9	79.9	43.1	96.9	
	RMS	0/1	76	74	75.6	14.7	77.6	42.5	96.9	0.252
		2/3	9	6	68.3	16.1	66.4	43.6	88.1	
	SAE	No	69	64	77.0	14.0	81.2	42.5	96.4	0.044
		Yes	23	23	70.6	14.8	67.8	43.1	96.9	
FACT-G	DLT	Yes	6	5	68.7	8.8	71.3	57.4	79.8	0.331
		No	86	81	74.0	14.4	75.0	28.7	94.4	
	CYCLE1 completed	Yes	62	58	73.6	14.5	75.0	28.7	94.4	0.959
		No	30	28	73.8	13.8	74.5	46.2	94.4	
	Replacement	No	80	76	73.4	14.3	75.0	28.7	94.4	0.697
		Yes	12	10	75.6	14.0	77.4	55.8	93.3	
	RMS	0/1	76	73	74.3	14.4	75.0	28.7	94.4	0.243
		2/3	9	6	67.9	14.0	63.3	55.6	85.2	
	SAE	No	69	63	74.9	14.5	79.6	28.7	94.4	0.190
		Yes	23	23	70.6	13.0	67.3	52.8	93.3	
MOSSSS	DLT	Yes	6	5	75.3	30.6	81.9	23.7	100.0	0.344
		No	86	81	87.8	15.9	93.4	19.7	100.0	
	CYCLE1 completed	Yes	62	58	85.9	18.9	92.1	19.7	100.0	0.977
		No	30	28	89.3	12.3	94.1	57.9	100.0	
	Replacement	No	80	76	85.9	17.7	92.1	19.7	100.0	0.155
		Yes	12	10	95.4	6.6	98.0	79.0	100.0	
	RMS	0/1	76	73	87.0	18.0	94.7	19.7	100.0	0.365
		2/3	9	6	84.7	13.9	88.8	61.8	100.0	
	SAE	No	69	64	87.1	16.4	92.8	19.7	100.0	0.659
		Yes	23	22	87.0	19.3	94.7	23.7	100.0	

FACT-G: Baseline Functional Assessment of Cancer Therapy—General Score. EORTC: Baseline European Organization for Research and Treatment of Cancer QLQ-C30 Score. MOSSSS: Baseline Medical Outcomes Study Social Support Survey Score. DLT: Dose limiting toxicity. RMS: Royal Marsden Score. SAE: Serious Adverse Event. N Obs *: Total number of patients. N **: Non-missing observations. ^: Mean and median reflect “N”.

**Table 4:** Comparison of Replacement vs non-Replacement groups in the seven studies that incurred subject replacement.

Variable	Statistic	Replacement		Overall	*p*-Value
		NO (n = 35)	YES (n = 12)		
FACT-G	Mean (SD)/N	70.9 (14.6)/34	75.6 (14)/10	72 (14.5)/44	0.419
	Median (Range)	70.2 (28.7, 93.3)	77.4 (55.8, 93.3)	72.6 (28.7, 93.3)	
EORTC	Mean (SD)/N	73.5 (15.1)/34	72.3 (20.9)/10	73.2 (16.3)/44	0.986
	Median (Range)	74 (42.5, 93.9)	79.9 (43.1, 96.9)	75.8 (42.5, 96.9)	
MOSSSS	Mean (SD)/N	83.8 (17.8)/34	95.4 (6.6)/10	86.4 (16.6)/44	0.041
	Median (Range)	90.1 (19.7, 100)	98 (78.9, 100)	91.4 (19.7, 100)	
CCI	Mean (SD)/N	8.2 (1.7)/35	9 (1.5)/12	8.4 (1.6)/47	0.121
	Median (Range)	8 (3, 11)	9 (6, 12)	9 (3, 12)	

FACT-G: Baseline Functional Assessment of Cancer Therapy—General Score. EORTC: Baseline European Organization for Research and Treatment of Cancer QLQ-C30 Score. MOSSSS: Baseline Medical Outcomes Study Social Support Survey Score. CCI: Baseline Charlson Comorbidity Index.

**Table 5:** Correlation of the different variables measured with Overall Survival.

Variable	Hazard Ratio (95% CI)	*p*-Value
FACT-G	0.96 (0.91, 1.01)	0.087
EORTC	0.97 (0.93, 1.01)	0.133
MOSSSS	0.98 (0.95, 1.02)	0.366
CCI	1.44 (0.89, 2.35)	0.142
RMS	2.70 (0.54, 13.52)	0.226
FACT-G group	3.12 (0.65, 15.04)	0.156
EORTC group	2.47 (0.62, 9.91)	0.201
MOSSSS group	1.47 (0.35, 6.16)	0.598

FACT-G: Baseline Functional Assessment of Cancer Therapy—General Score. EORTC: Baseline European Organization for Research and Treatment of Cancer QLQ-C30 Score. MOSSSS: Baseline Medical Outcomes Study Social Support Survey Score. CCI: Baseline Charlson Comorbidity Index. RMS: Baseline Royal Marsden Score 2/3 vs. 0/1. FACT-G group: Baseline FACT G Score <75 vs. >75. EORTC group: Baseline EORTC QLQ-C30 Score <77 vs. >77. MOSSSS group: Baseline Medical Outcomes Study Social Support Survey Score <93 vs. >93.

## Supplementary Materials

The following are available online at http://www.mdpi.com/2072-6694/9/7/73/s1. Table S1: Summary of 22 phase 1 trials; Table S2: Summary of 7 out of 22 phase 1 trials wherein subject replacement occurred; Table S3: Baseline Royal Marsden Score and outcomes; Table S4: EORTC QLQ C-30 questionnaire subcomponents and risk of SAE.
