# Supplementary material for: Quality-of-Life (QOL) during Screening for Phase 1 Trial Studies in Patients with Advanced Solid Tumors and Its Impact on Risk for Serious Adverse Events
Source: Cancers (Basel). 2017 Jun 26;9(7):73. doi: 10.3390/cancers9070073 (PMC5532609; doi:10.3390/cancers9070073)
Supplement: Supplementary file 1 [file cancers-09-00073-s001.pdf]

Supplementary Materials: Quality-of-Life (QOL) during Screening for Phase 1 Trial Studies in Patients with Advanced Solid Tumors and Its Impact on Risk for Serious Adverse Events

Sidra Anwar, Wei Tan, Chi-Chen Hong, Sonal Admane, Askia Dozier, Francine Siedlecki, Amy Whitworth, Ann Marie DiRaddo, Dawn DePaolo, Sandra M. Jacob, Wen Wee Ma, Austin Miller, Alex A. Adjei and Grace K. Dy

Table S1. Summary of 22 phase 1 trials.

Single-drug regimens (N = 11)	Combination regimens (N = 11)
Vaccine (N = 1)	Doublet cytotoxic chemotherapy (N = 1)
Cytotoxic chemotherapy (N = 1)	Signal transduction pathway inhibitor plus cytotoxic chemotherapy (N = 6)
Immunomodulatory (N = 2)	Signal transduction pathway inhibitor combination (N = 4)
Antibody-drug conjugate (N = 1)	
Signal transduction pathway inhibitor (N = 6)	

N: number of trials.

Table S2. Summary of 7 out of 22 phase 1 trails wherein subject replacement occurred.

	Regimen	N	Route of Drug Administration
**Single-drug regimens (N = 3)**	Cytotoxic chemotherapy	1	IV
	Immunomodulatory	1	IV
	Signal transduction pathway inhibitor	1	Oral
**Combination regimens (N = 4)**	Signal transduction pathway inhibitor plus cytotoxic chemotherapy	3	IV + Oral<br>IV + Oral<br>SQ + SQ
	Signal transduction pathway inhibitor combination	1	Oral + Oral

N: number of trials. IV: Intravenous. SQ: subcutaneous.

Table S3. Baseline Royal Marsden Score and outcomes.

Variable	Level	0/1	2/3	Overall	*p*-value
DLT	Yes	5 (6.6%)	1 (11.1%)	6 (6.5%)	0.500
	No	71 (93.4%)	8 (88.9%)	86 (93.5%)	
CYCLE1 completed	Yes	53 (69.7%)	5 (55.6%)	62 (67.4%)	0.456
	No	23 (30.3%)	4 (44.4%)	30 (32.6%)	
Replacement	No	68 (89.5%)	6 (66.7%)	80 (87%)	0.089
	Yes	8 (10.5%)	3 (33.3%)	12 (13%)	
SAE	No	57 (75%)	7 (77.8%)	69 (75%)	1.000
	Yes	19 (25%)	2 (22.2%)	23 (25%)	

DLT: Dose limiting toxicity, SAE: Serious Adverse Event.

Table S4. EORTC QLQ C-30 questionnaire subcomponents and risk of SAE.

EORTC QLQ C-30 Component ǂ	Variable	Level	N Obs *	N **	Mean ^	Standard Deviation	Median ^	Minimum	Maximum	*p*-value
Global Health Status	SAE	No	69	64	68.10	17.67	66.67	25.00	100.00	0.1143
		Yes	23	23	60.51	20.60	66.67	16.67	100.00	
Functional scales	SAE	No	69	64	77.91	15.80	82.50	36.67	100.00	0.0765
		Yes	23	23	71.90	16.29	71.67	41.67	100.00	
Symptom scales	SAE	No	69	64	22.75	13.80	20.80	0.00	59.62	0.0543
		Yes	23	23	29.23	14.70	30.77	1.92	53.85	

ǂ All of the scales and single-item measures range in score from 0 to 100. A higher score for the global health status QoL represents a higher QoL. A higher score for a functional scale represents a higher/healthier level of functioning. A higher score for a symptom scale/item represents a higher level of symptomatology/problems. SAE: Serious Adverse Event. N Obs *: Total number of patients. N **: Non-missing observations. ^ Mean and median reflect 'N'.

© 2017 by the authors; licensee MDPI, Basel, Switzerland. This article is an open access article distributed under the terms and conditions of the Creative Commons by Attribution (CC-BY) license (http://creativecommons.org/licenses/by/4.0/).
